# Fragment‐end sequence architecture of cell‐free DNA and its association with tumour DNA in non‐small cell lung cancer

**DOI:** 10.1002/ctm2.70901

**Published:** 2026-11-14

**Authors:** Dhruv Das, Prachi Gupta, Rahul Tambade, P. A. Hassan, A. V. S. S. Narayana Rao, Rajani Kant Chittela

**Affiliations:** ^1^ Applied Genomics Section Bioscience Group Bhabha Atomic Research Centre Mumbai India; ^2^ Homi Bhabha National Institute Mumbai India

**Keywords:** cell‐free DNA (cfDNA), circulating tumour DNA (ctDNA), fragment‐end signatures, fragmentomics, nanopore sequencing, non‐small cell lung cancer (NSCLC)

## Abstract

**Introduction:**

Circulating cell‐free DNA (cfDNA) fragmentation patterns provide a promising source of molecular information for liquid biopsy applications. However, the sequence features underlying fragmentation and their relationship to tumour‐derived DNA remain incompletely understood. Here, we characterized fragment‐end sequence signatures of extracellular cfDNA across cell lines, extracellular compartments, stress conditions and plasma from patients with non‐small cell lung cancer (NSCLC).

**Methods:**

cfDNA derived from cell lines was characterized using Oxford Nanopore sequencing, followed by fragment‐end analysis. Publicly available Nanopore cfDNA datasets from healthy individuals and NSCLC patients were analyzed to examine dinucleotide patterns and derive a motif ratio index. These signatures were further assessed for their association with estimated ctDNA abundance in an independent cohort of 50 EGFR‐mutant NSCLC patients.

**Results:**

Nanopore sequencing of A549, H460 and WI38 cell‐line cfDNA revealed reproducible end‐motif patterns, with enrichment of AT‐, GT‐ and CT‐containing termini. Analysis of plasma cfDNA from healthy individuals showed distinct end‐motif profiles from cell‐line‐derived cfDNA, with separation maintained after reducing 4‐mer motifs to 5′‐terminal dinucleotide features. An exploratory motif ratio based on these patterns showed a modest increase in lung cancer patients compared with healthy individuals. In an independent Illumina cohort of 50 NSCLC patients with activating EGFR mutations, 5′‐terminal dinucleotide frequencies were associated with estimated circulating tumour DNA (ctDNA) abundance using cross‐validated LASSO (Least Absolute Shrinkage and Selection Operator) regression (Spearman *ρ* = .73, *p* = 3.35 × 10^‒9^). The predefined motif ratio showed a strong correlation with estimated ctDNA concentration (Spearman *ρ* = .76, *p* = 1.89 × 10^‒10^).

**Conclusion:**

These findings suggest that simple, interpretable cfDNA end‐motif signatures capture sequence features associated with ctDNA abundance and warrant further validation for non‐invasive cancer monitoring.

## Highlights

- Cell‐line cfDNA showed consistent patterns in fragment‐end sequences.
- Healthy plasma cfDNA displayed distinct end‐motif patterns compared with cell‐line cfDNA.
- An exploratory motif ratio showed increased values in NSCLC patients compared with healthy individuals.
- cfDNA end‐motif signatures correlated with estimated ctDNA abundance in EGFR‐mutant NSCLC patients.

## Graphical abstract

Cell‐free DNA (cfDNA) fragmentation exhibits reproducible sequence‐dependent end signatures that remain stable across cell lines, extracellular compartments and stress conditions, despite substantial variation in cfDNA abundance and fragment size distributions. Using nanopore sequencing, we identified conserved fragment‐end motif patterns enriched for specific nucleotide patterns, indicating that cfDNA fragmentation reflects intrinsic cleavage preferences rather than random degradation. Analysis of plasma from healthy individuals and patients with non‐small cell lung cancer further revealed distinct dimer‐level signatures associated with tumour‐derived DNA. By integrating these dimer patterns, we developed a simplified motif ratio index that correlated with circulating tumour DNA abundance, supporting the utility of cfDNA fragment‐end signatures for non‐invasive cancer monitoring.

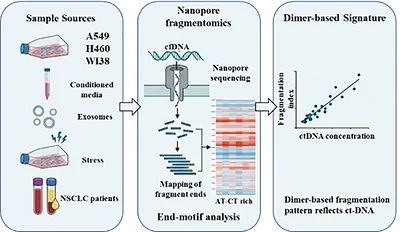

## 1 INTRODUCTION

Cell‐free DNA (cfDNA) has emerged as a critical biomarker in liquid biopsy applications^1^ particularly for non‐small cell lung cancer (NSCLC) detection and monitoring.^2^, ^3^, ^4^, ^5^ Circulating tumour DNA (ctDNA), which represents tumour‐specific genetic and epigenetic information in the cfDNA pool, can be detected via liquid biopsy.^6^, ^7^, ^8^ Analysis of cfDNA has therefore become an important tool for identifying actionable mutations, early detection, monitoring treatment response and tracking disease progression in NSCLC and other cancers.^9^, ^10^, ^11^, ^12^, ^13^ Beyond mutation detection, increasing evidence indicates that fragmentomic properties of cfDNA contain important biological information regarding their cellular origins and the mechanisms responsible for their release into circulation.^14^, ^15^, ^16^, ^17^, ^18^ Features such as fragment length distributions, nucleosome positioning patterns, genomic coverage biases and terminal sequence motifs reflect the interplay between chromatin organization and nuclease‐mediated DNA fragmentation during cellular DNA release.^15^, ^16^, ^19^, ^20^ These fragmentation patterns have been explored as complementary signals that may enhance the analytical power of cfDNA‐based assays.^21^, ^22^, ^23^, ^24^, ^25^, ^26^

Among fragmentomic features, the sequence composition at cfDNA fragment termini, commonly referred to as end motifs, has recently attracted considerable interest.^27^ These terminal nucleotide patterns are thought to arise from sequence preferences of nucleases involved in apoptotic DNA fragmentation, including enzymes such as DNASE1L3 and DFFB.^28^, ^29^, ^30^, ^31^, ^32^ Several studies have reported altered frequencies of specific end motifs in plasma cfDNA from cancer patients, suggesting that fragmentation‐associated sequence patterns may reflect underlying biological processes governing cfDNA generation.^16^, ^33^, ^34^

However, the biological interpretation and clinical utility of cfDNA end‐motif signatures remain incompletely understood. Many previous studies have relied on high‐dimensional motif representations, such as 4‐mer or longer nucleotide contexts, which capture detailed cleavage preferences but may be difficult to interpret and potentially sensitive to sequencing depth or assay design.^27^, ^31^, ^35^, ^36^, ^37^, ^38^, ^39^ Moreover, analyses performed directly on plasma samples often cannot easily disentangle intrinsic fragmentation biases from the heterogeneous mixture of DNA fragments originating from multiple cell types in circulation.^40^, ^41^, ^42^

In this study, we analyzed end‐motif patterns using controlled in vitro systems modeling DNA release to understand the sequence features underlying cfDNA fragmentation. Using 4‐mer end‐motif analysis, we captured detailed sequence patterns and subsequently represented these patterns at the 2‐mer level for simplification. Finally, the relevance of these features was assessed in lung cancer sequencing datasets. This approach bridges controlled experimental models with clinical data to study fragmentation‐associated sequence signatures.

## 2 RESULTS

### 2.1 cfDNA displays distinct fragmentation and conserved end‐motif signatures in vitro

Baseline fragmentation properties of extracellular cfDNA was studied from conditioned media of three human lung cell lines: A549, H460 and WI38. The isolated cfDNA varied in yield across cell lines (A549: 1.4 ± .08 ng/mL; H460: 7.14 ± .71 ng/mL; WI38: 1.79 ±  .15 ng/mL). Quantitative PCR (qPCR)‐based fragment integrity measurements showed higher DNA integrity in WI38 compared to A549 and H460 (Figure S1A). This was further supported by capillary electrophoresis profiles, which revealed enrichment of longer fragments (di‐ and tri‐nucleosomal) of cfDNA extracted from conditioned media of WI38, while cfDNA isolated from conditioned media of A549 and H460 exhibited relative enrichment of shorter fragments, with a higher proportion of mono‐nucleosomal cfDNA (Figure S1B).

To further examine these differences at single‐base resolution, direct profiling of individual cfDNA fragments was carried out using nanopore sequencing, facilitating the simultaneous assessment of fragment length and sequence composition. cfDNA samples extracted from the conditioned media of WI38 were relatively enriched for longer DNA fragments when compared to cfDNA samples derived from A549 and H460 (Figure 1A), consistent with the fragment integrity results and electrophoresis profiles. Long/short fragment ratios (151–250 bp/<151 bp) differed significantly across cell lines (*n* = 3 replicates per cell line; one‐way ANOVA, *p* < .001; Tukey's Honestly Significant Difference (HSD), *p* < .05 for all pairwise comparisons) (Figure 1B). Together, these findings suggest possible cell‐line‐specific differences in cfDNA release and fragmentation.

**FIGURE 1:**
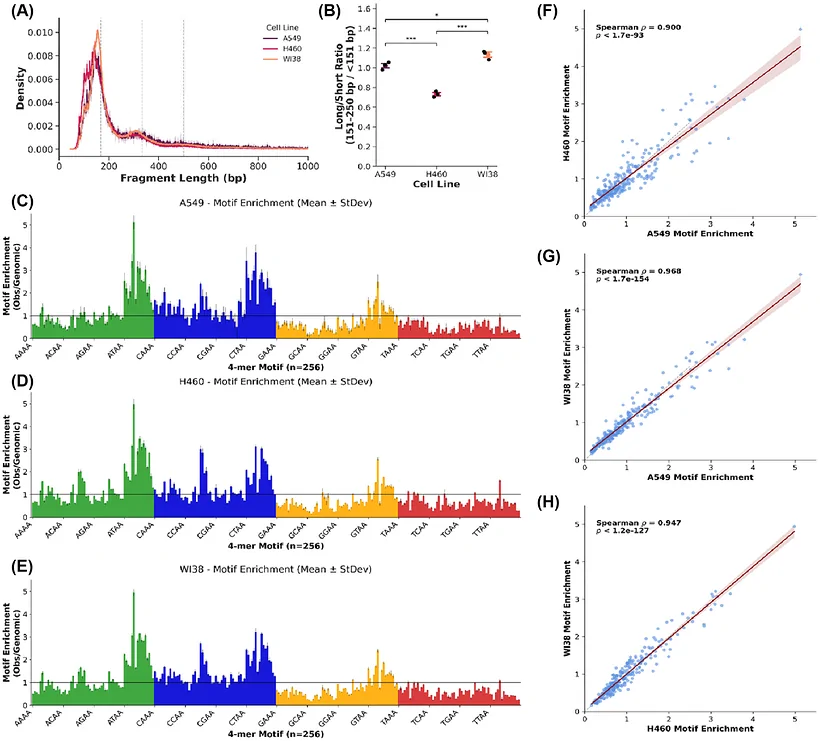
Baseline fragmentomic features of extracellular cell‐free DNA (cfDNA) derived from in vitro cell culture. (A) Fragment length distributions of cfDNA recovered from conditioned media of A549, H460 and WI38 cells, as assessed by nanopore sequencing, highlighting the predominance of short fragments characteristic of cfDNA (*n* = 3 replicates per group), with WI38 showing relative enrichment of longer cfDNA fragments. Solid lines represent group means with shaded bands indicating ±standard deviation (SD) across triplicates, and dashed grey vertical lines denote mono‐, di‐ and tri‐nucleosomal landmarks at 167, 334 and 501 bp, respectively. (B) Long/short fragment ratios (151‒250 bp/<151 bp) differ significantly across cell lines (*n* = 3 replicates per cell line; one‐way ANOVA *p* < .001). Data are presented as mean ± SEM, with distinct individual replicate values denoted by black points (Tukey's HSD post hoc test, *p* < .05 for all pairwise comparisons). ^*^ *p* < .05, ^***^ *p* < .0005. (C–E) Motif enrichment spectra of all possible 4‐mer sequences observed at the 5′ ends of cfDNA fragments in A549 (C), H460 (D) and WI38 (E) samples relative to an in silico generated genomic background derived from the GRCh38/hg38 reference genome. Motifs are colour‐coded by their first nucleotide, and frequencies are normalized within each sample. Values represent mean relative motif enrichment calculated across *n* = 3 replicates per cell line, with error bars denoting SD. The horizontal solid line at *y* = 1.0 indicates neutral expectation relative to genomic background. Ticks along the *x*‐axis are labelled at 16‐motif intervals. (F–H) Pairwise comparisons of 4‐mer motif enrichment ratios (observed/genomic frequency) between A549 versus H460 (F), A549 versus WI38 (G), and H460 versus WI38 (H). Each point represents a single 4‐mer motif plotted using the mean enrichment ratio across *n* = 3 replicates. Spearman rank correlation coefficients (*ρ*) indicate a strong concordance of end‐motif patterns across cell types. Red line indicates the linear trend, while the dotted line represents the line of identity (*y* = *x*).

5′ end motif preferences were assessed by calculating the frequencies of all possible 4‐mer sequences at the 5′ ends of the cfDNA fragments. The observed motif frequencies were then normalized using the corresponding frequencies derived from the (genomic) reference sequence. The end‐motif distributions showed clear deviations from genomic expectations (Figure 1C–E) with the motif enrichment and depletion patterns being consistent across A549, H460 and WI38 cell lines (Figure 1F–H; Spearman rank correlation coefficients, *ρ* = .900–.968). The pairwise Jensen–Shannon divergence (JSD) values between samples were low (.0031‒.0091), indicating comparable overall 4‐mer frequency distributions.

Ranking motifs by log_2_ enrichment relative to genomic background identified a subset of 4‐mers that were consistently either enriched across all samples (ATGT, CTCC, ATGA, ATCT, CTGA, ATCC and ATCA) or depleted (GCAG, TCAG, GCAC, GCCG and TTTT) (Figure S1C and Table S1). The observed enrichment of AT/CT‐rich ends and depletion of GC‐rich termini, suggest that fragmentation of cfDNA is likely influenced by non‐random, sequence‐dependent cleavage preferences.

To check whether sequencing depth differences might be affecting the observed cfDNA fragment length distribution and end‐motif results, a down‐sampling sensitivity analysis was performed. All replicates were normalized to match the depth of the lowest‐coverage sample (9710 reads), and fragment length distributions and motif enrichments were then compared before and after this correction. The fragmentation profiles of each cell line remained essentially unchanged after down‐sampling (Figure S1D). Motif enrichment was similarly stable with Spearman correlations between full‐depth and down‐sampled data ranged from  .98 to 1.00 across all replicates (Figure S1E). Analysis of the averaged log_2_ motif enrichments across cell lines confirmed that the enriched motifs (including ATGT, CTCC, ATGA, ATCT, CTGA, ATCC and ATCA) and depleted motifs (such as GCAG, TCAG, GCAC, GCCG and TTTT) maintained their distinct directional profiles across A549, H460 and WI38 (Figure S1F). These findings suggest that the observed fragmentation and end‐motif patterns are unlikely to be primarily driven by sequencing‐depth artefacts.

To rule out the possibility that the observed 4‐mer end‐motif enrichments were introduced as artefacts during library preparation or sequencing, the motif profile of WI38 genomic DNA (gDNA) was evaluated against cfDNA of WI38. Although gDNA exhibited a baseline correlation with cellular cfDNA (*ρ* ∼  .81) driven by overall genomic sequence composition, the substantial reduction in concordance compared to replicates (*ρ* > .98) demonstrates that the protocol itself does not universally impose these patterns (Figure S1G). Because both DNA sources underwent identical handling, the distinct, sequence‐specific terminal enrichments unique to cfDNA suggest that these motifs reflect molecular features of the circulating DNA pool rather than technical biases introduced during library preparation or sequencing.

To ensure our 4‐mer end‐motif findings were not driven by platform‐specific technical biases, we validated our results using single‐replicate Illumina sequencing datasets for each cell line (Figure S1H). Despite differences in sequencing technology, the 4‐mer motif profiles derived from the Illumina reads showed high concordance with our primary nanopore dataset (*ρ* > .90 across all cell lines). These findings indicate that the patterns are broadly reproducible across different sequencing systems, suggesting that end‐motif profiles are largely independent of the sequencing technology used.

Together, these results suggest that cfDNA fragmentation carries a consistent, biologically driven signature rather than random degradation. While the three cell lines showed differences in yield and fragment length, where cancer lines such as A549 and H460 released shorter fragments than the non‐tumourigenic WI38 line, all three shared a broadly similar pattern of 4‐mer motif enrichment and depletion, notably favouring AT‐ and CT‐rich motifs over GC‐rich ones. This signature remained stable across down‐sampling, stood out clearly from genomic background DNA, and showed strong concordance on an independent sequencing platform, pointing to genuine, sequence‐specific fragmentation patterns rather than technical bias.

### 2.2 cfDNA fragment size varies across extracellular compartments, while end‐motif patterns remain largely preserved

A considerable fraction of extracellular cfDNA was reported to be compartmentalized within exosomes^43^; however, findings across studies remain inconsistent.^44^ To determine if the extracellular context influences the fragmentomic features, we examined the cfDNA associated with exosome‐enriched (‘exo’) and exosome‐depleted supernatant (‘sup’) fractions derived from the cell lines. The exosome‐enriched fraction was characterized in A549 samples by nanoparticle tracking analysis (NTA), transmission electron microscopy (TEM), dynamic light scattering (DLS) analysis, and immunoblotting for canonical exosomal and non‐exosomal markers (Figure S2A–C). Vesicle diameters measured by TEM and NTA fell within the same size range expected for small extracellular vesicles (30‒200 nm). DLS reported a slightly larger hydrodynamic diameter (188 nm), which is expected due to hydration layer contributions and intensity‐weighted scattering biases. Furthermore, immunoblotting confirmed the presence of canonical markers (CD63 and TSG101) and the absence of the endoplasmic reticulum marker calnexin, providing additional evidence that the isolated fraction was enriched for exosomes.

cfDNA was subsequently extracted from both extracellular compartments of the three cell lines, and the absolute cfDNA abundance was quantified using qPCR (Figure S2D). Detectable cfDNA was present in both compartments across all cell lines. Higher cfDNA levels were observed in the exosome‐depleted supernatant for A549 and WI38 cells, whereas H460 displayed comparable abundance between the two fractions. Quantitative PCR‐based fragment integrity measurements showed a relative enrichment of longer DNA fragments in the exosome‐associated fraction compared with the supernatant (Figure S2E). Furthermore, single‐molecule nanopore sequencing of extracellular DNA extracted from conditioned media of A549 (*n* = 3) revealed distinct fragment size distributions between compartments (Figure 2A). Specifically, long/short fragment ratios (151–250 bp/<151 bp) differed significantly between the exosome‐associated cfDNA and exosome‐depleted supernatant fractions (*n* = 3 replicates per fraction; *t*‐test, *p* < .05), with exosome‐associated cfDNA enriched for longer fragments (Figure 2B). Consistent trends were also observed in cfDNA from H460 and WI38 (Figure S2H).

**FIGURE 2:**
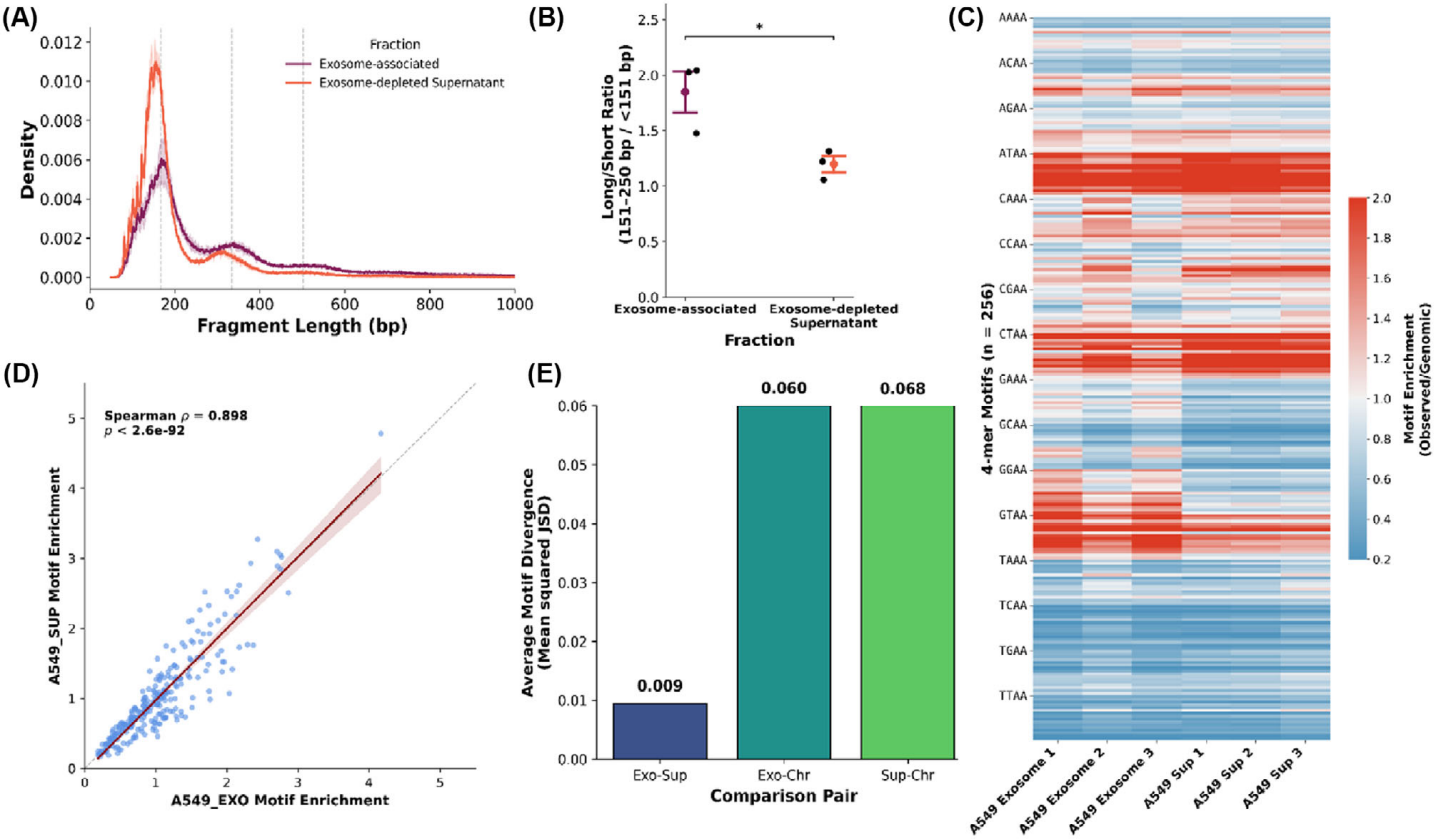
Extracellular compartmentalization influences cell‐free DNA (cfDNA) fragment size while preserving fragment‐end motif patterns in A549 cells. (A) Fragment length distributions of cfDNA isolated from exosome‐enriched (EXO) and exosome‐depleted supernatant (SUP) fractions of A549 (*n* = 3). Supernatant fractions are enriched for shorter DNA fragments, whereas exosome‐associated cfDNA exhibits broader fragment size distributions extending towards longer fragment lengths. (B) Long/short fragment ratios (151‒250 bp/<151 bp) differ significantly between the two compartments, demonstrating an enrichment of longer fragments in the exosome‐associated cfDNA fraction (A549, *n* = 3; *t*‐test *p* < .05). Data are presented as mean ± SEM, with distinct individual replicate values denoted by black. ^*^ *p* < .05. (C) Heatmap of normalized 4‐mer fragment‐end motif frequencies across all replicates of A549 origin. Motif architectures are largely conserved between extracellular compartments, with enrichment of specific terminal sequences, particularly motifs initiating with AT, CT and GT bases. (D) Scatter plots comparing motif enrichment ratios (relative to chromosomal background) between exosome‐associated and supernatant cfDNA for A549 (*n* = 3). Each point represents an individual 4‐mer motif. Strong concordance of motif enrichment patterns is observed across compartments (*ρ* = .90). Red line indicates the linear trend, while the dotted line represents the line of identity (*y* = *x*). (E) Jensen–Shannon divergence (JSD) values comparing fragment‐end motif distributions between sample groups. Exosome–supernatant comparisons exhibit low divergence (∼.009), indicating strong conservation of motif architecture across extracellular compartments of A549. In contrast, divergence between extracellular fractions and chromosomal background is higher.

Analysis of 4‐mer fragment end motifs further revealed comparable motif architecture across fractions (Figure 2C). In particular, enrichment of specific terminal sequences, especially motifs initiating with AT, CT and GT bases, was preserved across both exosome‐associated and exosome‐depleted fractions.

Motif enrichment profiles exhibited strong concordance between exosome‐associated and supernatant fractions across cell lines (A549: Spearman *ρ* = .90; H460: *ρ* = .83; WI38: *r* = .92) (Figures 2D and S2I,J). JSD values between exosome‐associated and supernatant fractions showed low divergence (∼.009), indicating strong conservation of fragment‐end motif architecture across extracellular compartments (Figure 2E). In contrast, the higher divergence between both extracellular fractions and the chromosomal background is consistent with a sequence‐dependent fragmentation signature in these fractions being distinct from the genomic baseline.

Differential analysis of 4‐mer end motifs between exosome‐associated and supernatant fractions (A549, *n* = 3) identified 22 motifs exhibiting nominal significance (*p* < .01, Table S2). Specifically, the exosome fractions showed higher levels of motifs starting with guanine, such as GGTA, GGCA and GGTT, while the supernatant fractions had more motifs starting with cytosine and thymine, such as CTGG and TTAT. The volcano plot reveals a clear pattern, almost all motifs enriched in the exosomes share guanine as their first base (Figure S2K). Although none of these motifs remained statistically significant after adjusting for multiple testing with false discovery rate (FDR) correction.

Collectively, these results demonstrate that extracellular vesicle associated and non‐vesicular cfDNA fractions differ in their fragment size distributions, with the former enriched for longer fragments. In contrast, fragment‐end motif architecture remains highly conserved between these compartments, despite subtle nominal enrichments of guanine‐starting motifs in the exosome fraction and cytosine‐ or thymine‐starting motifs in the supernatant.

### 2.2 Cellular stress alters cfDNA fragment length, while end‐motif patterns remain largely unchanged

Building on the observations that cfDNA fragment size varies across cell lines and extracellular compartments, while fragment‐end motif architecture is largely preserved, the role of altered cellular physiological conditions on the fragmentation features was investigated under serum deprivation. This is a well‐established stress condition known to perturb cellular homeostasis and modulate extracellular DNA release dynamics.^45^ To confirm that this stress induction altered the cellular state, Annexin V‐FITC/PI flow cytometry and lactate dehydrogenase (LDH) release assays were performed in A549 cells (*n* = 3). Flow cytometry revealed detectable, although low‐level, cell death upon serum withdrawal, with viable cells shifting from 91.17 ± .40% in controls to 83.93 ± .51% under serum‐free conditions, alongside modest increases in apoptotic and late‐stage damaged fractions (Figure S3A). Consistently, LDH release assay indicated a low‐level cytotoxic response upon serum withdrawal (8.17 ± 3.55%), whereas the positive control (cisplatin) elicited a substantially higher response (26.99 ± 2.18%) (Figure S3B).

Serum deprivation led to a marked increase in extracellular cfDNA abundance across all cell lines compared with serum‐containing conditions (complete medium [CM] and serum‐deprived [DMEM] samples respectively, Figure S3C; paired *t*‐test, all *p* < .005). Long/short fragment ratios (151–250 bp/<151 bp) showed an increase in DMEM cell culture supernatant compared to CM controls in A549 cells (*n* = 3), with DMEM‐derived cfDNA exhibiting a higher proportion of longer fragments, although the difference did not reach statistical significance (Figure 3A). Similar increases in fragment length distributions were also observed in H460 and WI38 cells under serum deprivation (Figure S3D).

**FIGURE 3:**
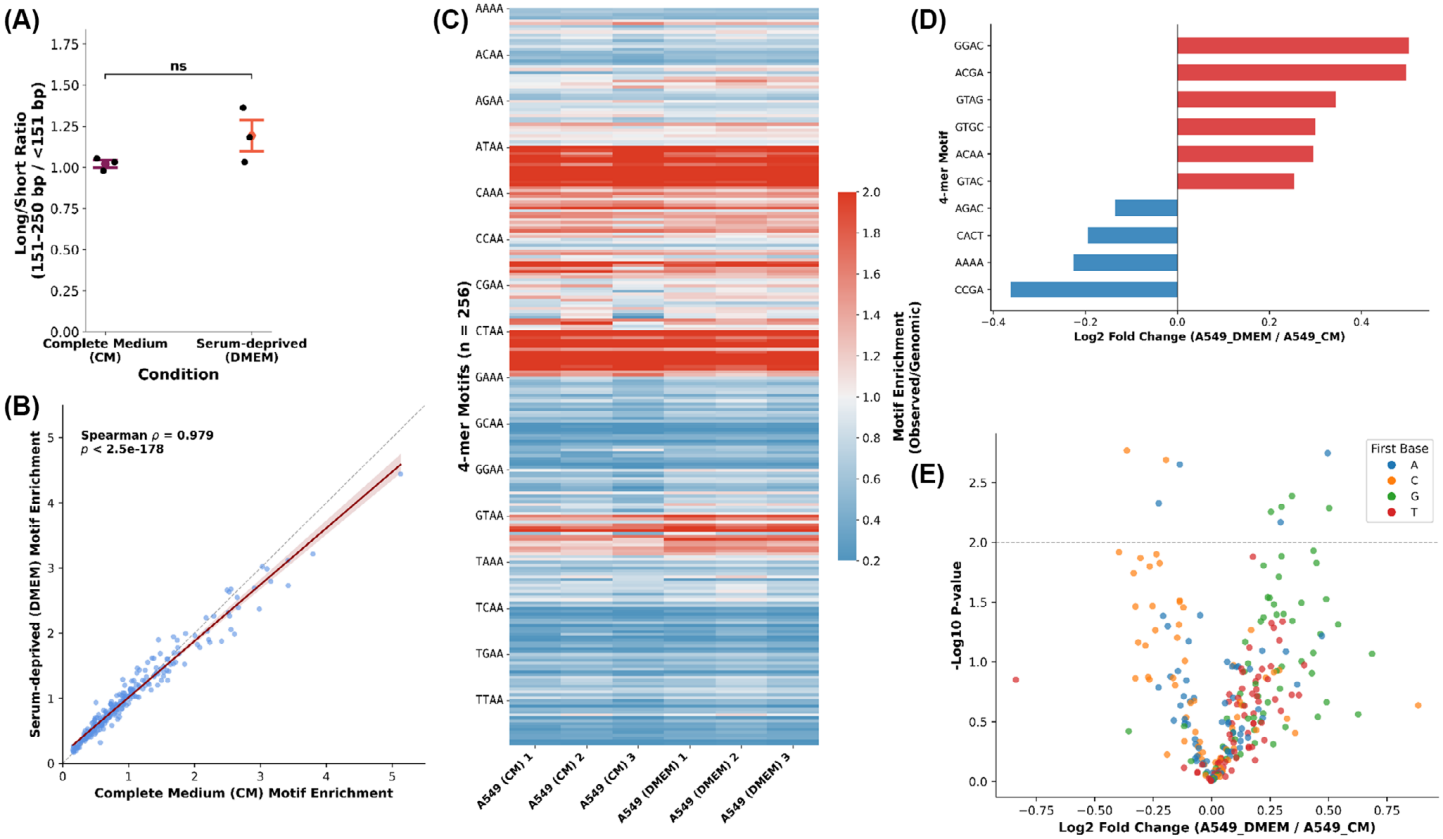
Serum deprivation shifts fragment size distributions in A549 cells while preserving core end‐motif architecture. (A) Long/short fragment ratios (151–250 bp/<151 bp) compared between complete medium (CM) and serum‐deprived (DMEM) culture conditions (A549, *n* = 3). Data are presented as mean ± SEM, with individual replicate values denoted by black points. (B) Pairwise comparison of normalized 4‐mer fragment‐end motif frequencies between serum‐containing (CM) and DMEM conditions for A549 cells. Each point represents an individual 4‐mer motif. A strong concordance between conditions (*ρ* = .98, *p* < 1 × 10^‒90^) indicates that fragment‐end motif composition remains highly stable. Red line indicates the linear trend, while the dotted line represents the line of identity (*y* = *x*). (C) Heatmap of normalized 4‐mer fragment‐end motif frequencies. Motif architectures are largely conserved between CM and DMEM conditions (A549, *n* = 3), showing consistent enrichment of specific terminal sequences, particularly motifs initiating with AT, CT and GT bases. (D) Top nominally significant 4‐mer end motifs (*p* < .01) between CM and DMEM conditions. Horizontal bar chart depicting the log_2_ fold change (DMEM/CM) of the 10 motifs identified in A549 cells (*n* = 3). Positive values indicate higher enrichment in DMEM conditions, while negative values indicate higher enrichment in CM. None of these motifs remained statistically significant after false discovery rate correction (*q* > .05). (E) Volcano plot of differential 4‐mer end‐motif enrichment between CM and DMEM conditions. Scatter plot displaying the log_2_ fold change (DMEM/CM) against log_10_ *p*‐value for all 256 4‐mer motifs in A549 cells (*n* = 3). Points are coloured by their first nucleotide base (blue: A, orange: C, green: G, red: T). The horizontal dashed line marks the nominal significance threshold (*p* = .01).

Motif enrichment profiles exhibited strong concordance between CM and DMEM culture conditions across cell lines (A549: Spearman *ρ* = .98; H460: *ρ* = .86; WI38: *r* = .91) (Figures 3B and S3E,F).

Analysis of 4‐mer fragment end motifs further demonstrated comparable motif architecture across conditions (Figure 3C). Specifically, the enrichment patterns of terminal sequences initiating with AT, CT and GT bases remained consistent across CM and DMEM culture environments.

Differential analysis of 4‐mer end motifs between CM and DMEM culture conditions (A549, *n* = 3) identified 10 motifs exhibiting nominal significance (*p* < .01) (Figure 3D). Specifically, CM‐derived samples showed higher enrichment for select motifs such as CCGA (log_2_ fold change [FC] = ‒.36), whereas DMEM conditions exhibited higher levels for motifs such as ACGA (log_2_FC = .50) and GGAC (log_2_FC = .50). The volcano plot illustrates the distribution of these minor shifts across nucleotide start bases (Figure 3E). Consistent with the global preservation of motif architecture, none of these differentially enriched motifs remained statistically significant after adjusting for multiple testing with FDR correction (*q* > .05).

Overall, while serum deprivation increases extracellular cfDNA levels and slightly alters fragment size distributions, the core 4‐mer end‐motif profiles remain largely consistent. The strong overall correlation across cell lines and the absence of significant differences after multiple testing correction indicate that baseline fragment‐end features are largely preserved under these conditions.

### 2.3 Fragment‐end motif architecture of cfDNA in healthy and NSCLC plasma samples

To examine whether the patterns observed in the cell‐line experiments were also present in circulating cfDNA, we analyzed publicly available nanopore sequencing data of plasma cfDNA from seven healthy individuals (H01‐H07), obtained from the NCBI Sequence Read Archive (SRA; BioProject PRJNA768481) and originally generated in a previously published study.^46^ We compared their end‐motif profiles with those of cfDNA derived from cell lines (A549, H460 and WI38). We calculated the relative frequency of all 256 possible 4‐mer end motifs in each sample to compare the end‐motif patterns between healthy plasma cfDNA and cfDNA derived from the cell lines. Cell lines and healthy individuals showed distinct motif enrichment signatures, each highly consistent within its group, which clearly separated the two sample types (Figure 4A). To assess global variance and structure in 4‐mer end‐motif frequencies without supervision, we performed principal component analysis (PCA) across all samples. Cell‐line‐derived cfDNA and healthy plasma cfDNA formed distinct clusters (Figure 4B). PC1 explained 71.7% of the total variance and clearly separated the two groups, suggesting that their end‐motif profiles differed systematically.

**FIGURE 4:**
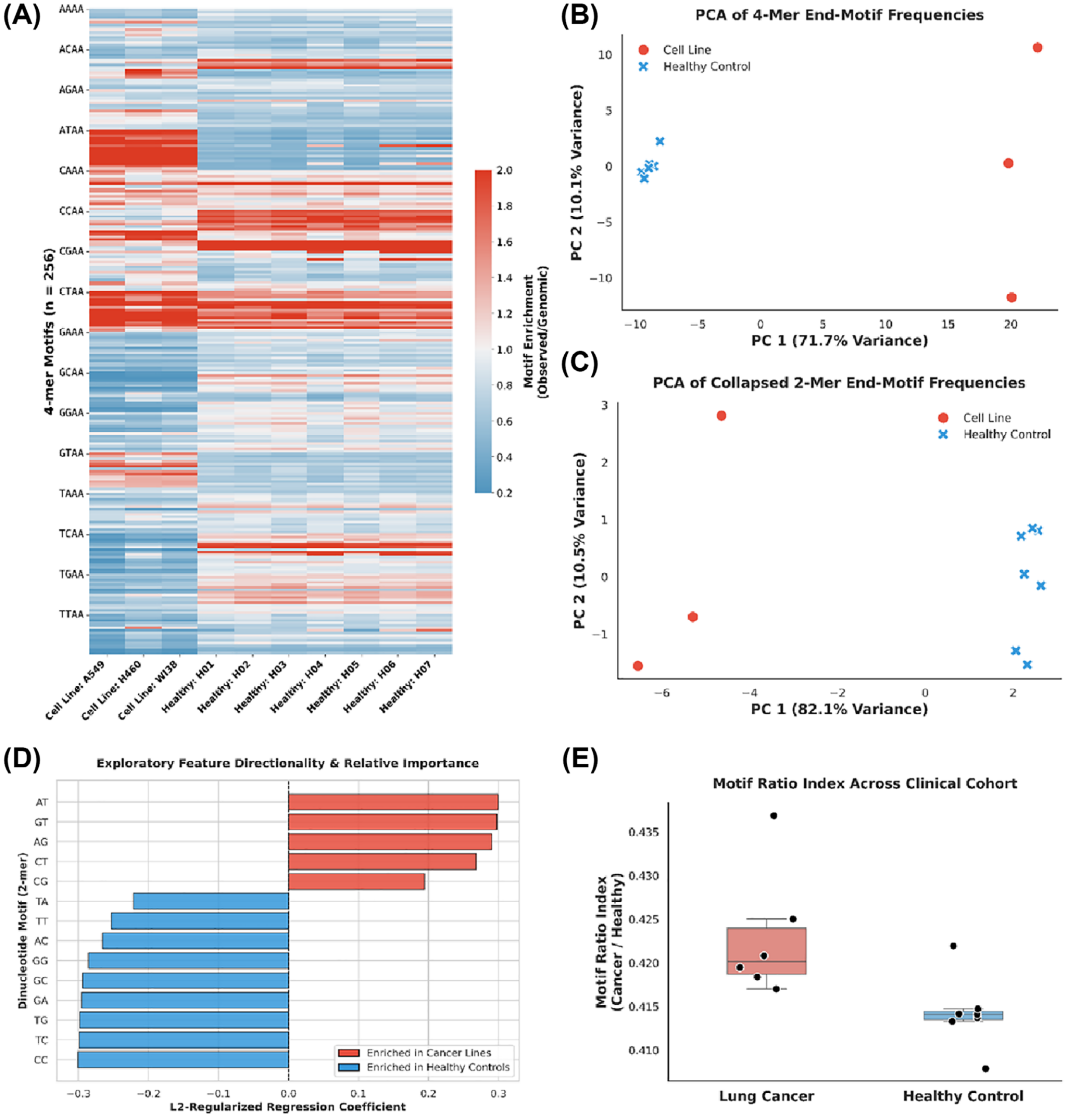
Distinctive end‐motif enrichment signatures and predictive profiles differentiate cell lines and lung cancer patients from healthy controls. (A) Heatmap of normalized 4‐mer frequencies profiles across cell lines and healthy control samples. Cell lines and healthy individuals display distinct, highly consistent motif enrichment signatures. (B) Principal component plot based on 4‐mer end‐motif frequency profiles, with PC 1 accounting for 71.7% of the total variance and PC 2 accounting for 10.1%. Red circles represent cell‐line samples and blue crosses represent healthy controls. (C) Principal component plot based on dinucleotide (2‐mer) end‐motif frequency profiles, with PC 1 accounting for 82.1% of the total variance and PC 2 accounting for 10.5%. Red circles represent cell‐line samples and blue crosses represent healthy controls. Bar plot of the top enriched 4‐mer motifs ranked by mean log_2_ enrichment relative to genome‐wide background frequencies. Cytosine‐rich motifs, including CCTT, CCTA, CCCA and CCAT, show consistent enrichment at fragment termini, indicating preferential fragmentation in specific sequence contexts. (D) Horizontal bar plot of L2‐regularized regression coefficients showing the relative contribution and directionality of dinucleotide (2‐mer) end motifs in distinguishing cell lines from healthy controls. Red bars indicate positive coefficients for motifs enriched in cell lines (e.g., AT, GT, AG), while blue bars indicate negative coefficients for motifs enriched in healthy controls (e.g., CC, TC, TG). (E) Box plot comparing the motif ratio index (cancer/healthy motifs) between patients with lung cancer (*n* = 6) and healthy controls (*n* = 7), with individual sample data points overlaid as black dots. Boxes show the median and interquartile range (IQR).

To determine whether the observed separation was maintained at a simpler level of analysis, we grouped the 4‐mer end‐motif frequencies into the 16 possible 5′‐terminal dinucleotide frequencies and repeated the PCA. Cell‐line‐derived and healthy plasma cfDNA samples again formed two distinct, non‐overlapping clusters along PC1, which explained 82.1% of the total variance (Figure 4C). The persistence of this separation after grouping the motifs suggests that the dinucleotide representation captures a substantial component of the variation observed at the 4‐mer level, while providing a simpler representation of fragment‐end sequence composition. To identify the motifs contributing to this separation, we compared the frequencies of the 2‐mer motifs between the two groups. Since the cell‐line group was small, we used the non‐parametric Mann‒Whitney *U*‐test with Benjamini‒Hochberg correction for multiple testing. We also used an exploratory L2‐regularized linear model based on the 2‐mer frequencies to assess the direction and relative contribution of each dinucleotide to the group difference. In this model, positive coefficients indicated enrichment in cell‐line‐derived cfDNA, including AT, GT, AG, CT and CG, whereas negative coefficients indicated enrichment in healthy plasma cfDNA, including CC, TC, TG, GA, GC, GG, AC, TT and TA (Figure 4D).

To summarize these feature‐level differences in a single metric, we defined an exploratory motif ratio index: the summed frequency of the five dinucleotides enriched in cell‐line‐derived cfDNA (AT, GT, CT, CG and AG) divided by the summed frequency of the nine dinucleotides enriched in healthy plasma cfDNA (CC, AC, TA, TT, GG, GC, GA, TG and TC). To assess whether the motif ratio index defined from the cell‐line comparison could also distinguish lung cancer from healthy plasma cfDNA, we calculated the index for six lung cancer patients and the same seven healthy individuals using data from the same published study. The index was modestly higher in the lung cancer group than in the healthy group (Figure 4E), with a trend towards separation at the individual sample level. Although these findings are exploratory, they suggest that the end‐motif patterns observed in cell‐line‐derived cfDNA may capture features that are also present in circulating cfDNA in lung cancer.

### 2.4 Dimer‐level fragmentation signatures associate with ctDNA abundance in NSCLC plasma samples

We next asked whether end‐motif patterns were associated with ctDNA abundance in patients with NSCLC. We analyzed Illumina short‐read whole‐genome plasma cfDNA sequencing data from 50 NSCLC patients carrying activating EGFR driver mutations, including exon 19 deletions and L858R. We estimated ctDNA concentration by multiplying the variant allele fraction (VAF) of the tumour‐associated EGFR mutation by the corresponding total plasma cfDNA concentration, providing an approximate measure of the tumour‐derived fraction of circulating cfDNA (Table S3). Across the 50 patients, the median plasma cfDNA concentration was  .445 ng/mL, while the estimated median ctDNA concentration was  .021955 ng/mL.

To determine whether end‐motif composition could predict ctDNA abundance, we fitted an L1‐regularized linear regression model (LASSO) using the frequencies of all 16 5′ dinucleotides as predictors and log_10_‐transformed ctDNA concentration as the outcome. Model performance was evaluated using fivefold cross‐validation, with each patient's ctDNA value predicted by a model that did not include that patient during training. The predicted and observed ctDNA concentrations showed a strong correlation (Pearson *r* = .72, *p* = 3.35 × 10^‒9^; Spearman *ρ* = .73, *p* = 2.05 × 10^‒9^; Figure 5A), indicating that 5′ dinucleotide end‐motif patterns capture information associated with ctDNA abundance.

**FIGURE 5:**
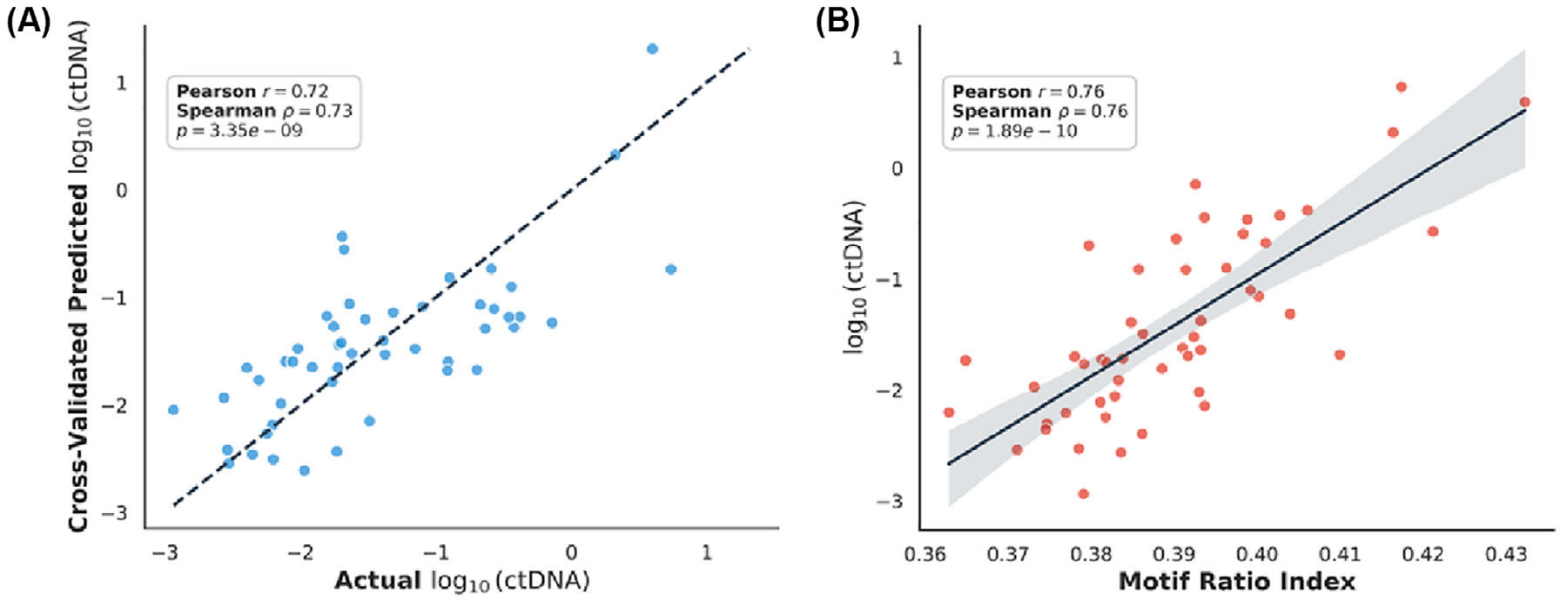
Dimer‐level fragmentation signatures reflect circulating tumour DNA (ctDNA) abundance in non‐small cell lung cancer (NSCLC) plasma samples. (A) Scatter plot comparing actual log_10_ (ctDNA) fraction against cross‐validated predicted log_10_ (ctDNA) fraction, with individual samples shown as light blue dots and the identity line (*y* = *x*) represented as a dashed diagonal line. Statistical correlation is indicated by Pearson *r* = .72 and Spearman *ρ* = .73 (*p* = 3.35 × 10^−9^). (B) Scatter plot showing the linear relationship between the motif ratio index (*x*‐axis) and log_10_ (ctDNA) fraction (*y*‐axis), with individual samples plotted as red points, a linear regression trend line (dark line), and the 95% confidence interval shaded in grey. Correlation metrics are indicated by Pearson *r* = .76 and Spearman *ρ* = .76 (*p* = 1.89 × 10^−10^).

We then asked whether this association could be captured using the motif ratio defined in our earlier analysis. The motif ratio was calculated for each NSCLC sample using the 5′‐terminal dinucleotide frequencies from the Illumina sequencing data, without changing the motifs or their definition established from the independent nanopore dataset. The motif ratio showed a strong positive correlation with estimated ctDNA concentration (Pearson *r* = .76, *p* = 1.89 × 10^‒10^; Spearman *ρ* = .76, *p* = 2.32 × 10^‒10^; Figure 5B). Notably, this simple motif ratio showed a correlation comparable to that of the cross‐validated LASSO model, despite using only a predefined combination of 5′‐terminal dinucleotides. These findings suggest that the identified motif ratio index captures a substantial part of the end‐motif signal associated with ctDNA abundance.

Taken together, these findings show that 5′‐terminal dinucleotide patterns are associated with ctDNA abundance in plasma. The motif ratio index defined from the independent nanopore analysis retained a strong correlation with estimated ctDNA concentration in an independent Illumina NSCLC cohort, suggesting that this simple end‐motif measure may provide a useful approach for assessing the tumour‐derived component of circulating cfDNA.

## 3 DISCUSSION

Circulating cfDNA fragmentation patterns provide a molecular footprint of DNA release and degradation processes in vivo.^18^, ^47^ Here, we systematically characterized fragment length and fragment‐end sequence signatures of extracellular cfDNA across multiple contexts, including cultured cell lines, exosome‐associated fractions, physiologically perturbed conditions and plasma from NSCLC patients. Our analyses reveal that cfDNA fragmentation exhibits reproducible, sequence‐dependent fragment‐end motif patterns that vary with the biological source of cfDNA and can be summarized at the dinucleotide level. These patterns were associated with tumour‐derived DNA abundance in plasma, suggesting that fragment‐end sequence features may provide information about the tumour‐derived component of circulating cfDNA.

Nanopore sequencing allowed us to examine cfDNA molecules across a broad range of fragment sizes without setting a predefined size range, giving a more unbiased view of the cfDNA length distribution. It also retained sequence information from individual fragments, allowing fragment length to be examined together with sequence‐derived features such as end motifs. Using single‐molecule nanopore sequencing, we first established baseline fragmentation characteristics of extracellular cfDNA from cultured A549, H460 and WI38 cells. Despite differences in total DNA abundance and fragment length distributions, fragment‐end motif architecture was remarkably consistent, with enriched 4‐mer motifs frequently containing AT or CT prefixes, whereas GC‐rich motifs were depleted. These findings indicate that cfDNA fragmentation is not random but reflects intrinsic sequence‐dependent cleavage preferences. Such biases likely arise from the sequence‐specific activity of circulating and intracellular nucleases, consistent with prior observations that nuclease preferences strongly influence cfDNA fragment‐end composition.^30^, ^31^, ^32^, ^34^

We next assessed the influence of extracellular compartmentalization and cellular stress. Comparison of exosome‐associated cfDNA with supernatant DNA revealed differences in fragment length distributions, with exosomal DNA enriched for longer fragments, yet the motif architecture remained largely unchanged. Similarly, serum deprivation as a stress condition increased cfDNA release and altered fragment size distributions but produced minimal changes in end‐motif composition. Together, these results suggest that while cfDNA concentration and fragment length are sensitive to cellular physiology and the extracellular context, end‐motif composition is relatively stable across these conditions. Using tetramer and dimer abundance analysis, we concluded that a substantial portion of the motif‐level signal could be captured at the dimer level. This reduction in sequence complexity provided a basis for developing a simpler and more interpretable representation of fragment‐end composition.

Fragment length, nucleosome positioning, and methylation capture different aspects of cfDNA biology. While fragment length and nucleosome positioning provide information about DNA size and chromatin organization, the motif ratio reflects sequence‐dependent patterns at fragment ends. Similarly, methylation‐based markers provide information about tissue origin and epigenetic state that is not captured by the motif ratio. Therefore, the motif ratio is not intended to replace these features, but may provide a simple and interpretable measure that could complement them. Combining these features may provide a more comprehensive view of cfDNA and improve its utility for cancer classification and monitoring.

Extending these observations to clinical samples, cfDNA from NSCLC patient plasma showed a distinct end‐motif profile compared with cfDNA derived from cell lines. Healthy plasma cfDNA was enriched for several dinucleotides, including CC, GC, GA and GG, whereas AT and CT were more prominent in cell‐line‐derived cfDNA. PCA showed that these differences were retained after reducing the analysis from 4‐mer to dinucleotide resolution, indicating that the separation was not driven by only a small number of individual 4‐mers.

These patterns differ from cell‐line‐derived extracellular DNA, reflecting the complex in vivo environment influenced by contributions from multiple tissue and the combined effects of intracellular and extracellular nucleases. Thus, although the basic end‐motif architecture was conserved, its relative composition differed between cell‐derived and circulating cfDNA.

Although the cell‐line experiments provide a controlled system for characterizing cfDNA end‐motif patterns, the in vivo plasma environment is considerably more complex. The differences in end‐motif composition between cell‐line‐derived and plasma cfDNA may reflect a combination of biological factors, including cellular and tissue origin, chromatin organization, and nuclease activity. In plasma, cfDNA is derived from multiple cellular sources, and the contribution of tumour‐derived DNA may therefore vary across samples. Differences in sample processing and sequencing platforms may also influence the observed motif frequencies. Together, these factors may contribute to the distinct end‐motif profiles observed between cell‐line‐derived and plasma cfDNA. The detection of related motif patterns in plasma nevertheless suggests that some sequence‐dependent features may be retained in circulation, although their biological origin and relative contribution require further investigation.

To capture these differences in a simple measure, we developed a motif ratio index based on dinucleotides enriched in cell‐line‐derived cfDNA relative to those enriched in healthy plasma cfDNA. Importantly, the motif groups used to define this ratio were established from the initial analysis and were not re‐optimized in the NSCLC cohort. When applied to an independent set of Illumina whole‐genome sequencing data from 50 patients with EGFR‐mutant NSCLC, the motif ratio showed a strong correlation with estimated ctDNA concentration. his association was comparable to that obtained using an L1‐regularized LASSO model based on all 16 dinucleotides, which showed a Pearson correlation of  .72 with the observed ctDNA estimates, while the motif ratio showed a Pearson correlation of  .76. The ability of a simple predefined ratio to capture a signal similar to that obtained from a multivariable model suggests that a substantial part of the information associated with estimated ctDNA abundance can be represented using a small number of end‐motif features.

Mechanistically, the observed sequence biases likely reflect the interplay of chromatin organization and nuclease activity.^29^, ^48^ Apoptotic and necrotic DNA cleavage is influenced by nucleosome positioning and accessibility, which impose sequence‐dependent constraints on fragmentation, while circulating nucleases further shape fragment ends.^18^ The consistent enrichment of specific motifs across different biological samples suggests that these processes generate reproducible fragmentation patterns and can serve as molecular fingerprints of cfDNA release.^27^, ^35^ However, our data do not directly establish the contribution of individual nucleases, chromatin structure, or other molecular processes to the observed sequence biases. The mechanistic basis of these end‐motif patterns therefore remains to be determined.

The findings of this study indicate that dimer‐based fragmentomic signatures provide biologically informative measures associated with tumour‐derived DNA abundance in plasma. Several limitations should be considered when interpreting these findings. First, the initial motif patterns were derived from a relatively small number of cell lines and healthy plasma samples, and cell‐line‐derived cfDNA cannot fully reproduce the complexity of cfDNA release and clearance in patients. Second, the motif ratio was defined using nanopore‐derived data and subsequently applied to Illumina sequencing data. Differences in sequencing chemistry, library preparation and error profiles between platforms may influence end‐motif frequencies. In particular, nanopore sequencing can exhibit systematic base‐calling errors, especially in homopolymer and GC‐rich regions, which may affect single‐base‐resolution analysis of fragment ends. Independent Illumina sequencing of the cell‐line samples showed high concordance with the primary nanopore‐derived motif profiles (Spearman *ρ* > .90), supporting the reproducibility of the observed end‐motif patterns across platforms. Nevertheless, because the cell‐line and plasma datasets were generated from different biological sources and sequencing workflows, residual batch effects cannot be completely separated from biological differences between the sample types. These technical factors should therefore be considered when interpreting differences in motif composition. Importantly, the predefined motif ratio showed a consistent association with estimated ctDNA abundance when applied to the independent Illumina cohort, although the contribution of platform‐specific effects cannot be completely excluded. Third, ctDNA concentration was estimated from EGFR mutation VAF and total plasma cfDNA concentration rather than measured directly, and therefore represents an indirect estimate of tumour‐derived DNA abundance. This approximation may be influenced by copy‐number alterations, tumour heterogeneity, and mutation‐specific differences in ctDNA shedding, which may affect the relationship between VAF and the actual amount of tumour‐derived DNA. Therefore, the observed associations should be interpreted as relationships with estimated ctDNA abundance rather than direct measurements of ctDNA concentration. Finally, the cohort included only NSCLC patients with activating EGFR mutations, and broader validation across different molecular subtypes and larger independent cohorts is required.

The exploratory analysis of publicly available plasma cfDNA data from healthy individuals and lung cancer patients also showed differences in motif ratio values between the two groups. However, the small sample size limits any assessment of diagnostic performance, and these observations should therefore be considered hypothesis generating rather than evidence of diagnostic utility. Similarly, the current findings support an association between end‐motif composition and ctDNA abundance but do not establish that the motif ratio can directly quantify ctDNA. Future studies incorporating larger, multi‐cancer cohorts, independent and clinically diverse cohorts and additional fragmentomic features—such as fragment size profiles, nucleosome positioning and methylation—may enhance the predictive power of sequence‐based signatures and their utility for non‐invasive cancer monitoring. Direct measurements of ctDNA, longitudinal sampling and validation across sequencing platforms will also be important to determine whether these signatures can provide robust measures of tumour burden and treatment response. Overall, these findings support the potential of simplified cfDNA end‐motif signatures as interpretable features of tumour‐derived DNA, while highlighting the need for further clinical validation.

## 4 CONCLUSION

In conclusion, our findings show that cfDNA fragmentation exhibits reproducible, sequence‐dependent end‐motif patterns that can be represented using simple and interpretable dimer‐level signatures. A motif ratio defined from these patterns showed a strong association with estimated ctDNA abundance in an independent NSCLC cohort, indicating that simplified end‐motif signatures can capture a substantial part of the sequence‐based signal associated with tumour‐derived cfDNA. These findings support the potential of cfDNA end‐motif features as informative components of liquid biopsy approaches, while further validation in larger and clinically diverse cohorts is needed to establish their clinical utility.

## 5 MATERIALS AND METHODS

### 5.1 Cell culture, conditioned media collection and cfDNA extraction

Human lung cancer cell lines A549 and H460, included as widely used models representing distinct NSCLC subtypes, and the non‐malignant lung fibroblast cell line WI38, were cultured in Dulbecco's Modified Eagle Medium (HiMedia, Cat#AL007G) supplemented with 10% foetal bovine serum (Gibco, Cat#A52567‐01) and 1% penicillin–streptomycin (HiMedia, Cat#A001A). Cells were maintained at 37°C in a humidified incubator with 5% CO_2_.

For extracellular DNA collection, 5 × 10^6^ cells were seeded in 150 mm cell culture dish and allowed to attach for 24 h, after which the culture medium was replaced with the indicated medium. Following an additional 24 h incubation, conditioned media were collected. Cells were maintained at ∼70%–80% confluency at the time of collection. Conditioned media were collected and centrifuged at 1000 × *g* for 5 min followed by 10 000 × *g* for 10 min to remove cells, apoptotic bodies and debris. The clarified supernatant was stored at −80°C until further processing.

cfDNA was extracted from 5 mL of conditioned media and 1 mL of exosomal fractions using the QIAamp Circulating Nucleic Acid Kit (Qiagen, Cat#55114) according to the manufacturer's instructions. DNA was eluted in nuclease‐free water. Each experiment was independently repeated three times unless otherwise mentioned.

gDNA was extracted from approximately 1 × 10^6^ WI38 cells using the GenElute Mammalian Genomic DNA Miniprep Kit (Sigma‒Aldrich, Cat#G1N350) according to the manufacturer's instructions.

### 5.2 Isolation and characterization of extracellular vesicles

Conditioned media were collected and sequentially centrifuged at 1000 × *g* for 5 min and 10 000 × *g* for 30 min to remove cells, apoptotic bodies, debris and larger vesicles. The clarified supernatant was subjected to ultracentrifugation at 100 000 × *g* for 90 min at 4°C (Hitachi, CS120FNX). The resulting pellet was collected as the exosome‐enriched (exo) fraction, while the supernatant was retained as the exosome‐depleted (sup) fraction. The exosome pellet was washed once with phosphate‐buffered saline (PBS; Sigma, Cat#P5493) and re‐pelleted under the same conditions. Both fractions were either used immediately or stored at −80°C until further processing.

Extracellular vesicles were characterized using NTA (NanoSight NS300, Malvern Instruments) to determine size distribution.

DLS measurements were performed to determine the hydrodynamic diameter of the isolated exosomal fraction. Exosome pellets were resuspended in filtered PBS, loaded into a disposable cuvette, and analyzed using a Litesizer DLS 500 at 25°C to determine hydrodynamic diameter and size distribution.

Vesicle morphology was assessed by TEM. Briefly, exosome‐enriched fractions suspended in PBS were applied onto carbon‐coated copper grids. After adsorption, grids were washed with PBS and negatively stained with 2% uranyl acetate. Excess stain was removed, and grids were washed with 20%, 40%, 60%, 80% and finally with absolute ethanol, air‐dried prior to imaging. Samples were visualized using a transmission electron microscope (Zeiss Libra 120) operated at an accelerating voltage of 80–120 kV.

Presence of exosome markers (CD63 and TSG101) alongside the absence of the endoplasmic reticulum marker calnexin in the preparations were confirmed by Western blotting. Protein lysates were prepared and equal amounts of protein were separated by SDS–PAGE (Sodium Dodecyl Sulfate–Polyacrylamide Gel Electrophoresis) and transferred onto PVDF (Polyvinylidene Fluoride) membranes. Membranes were blocked and incubated with primary antibodies against CD63 (Sigma, Cat#SAB5700799; 1:500) and TSG101 (Sigma, Cat#T5701; 1:500), and calnexin (Cell Signaling Technology, Cat#2679T; 1:1000) followed by HRP‐conjugated secondary antibodies (Cell Signaling Technology, Cat#7074P2; 1:5000). Signals were detected using chemiluminescence (ECL) and imaged using a chemiluminescence detection system.

### 5.3 Annexin V‐FITC/propidium iodide flow cytometry assay

Cell viability and apoptosis were evaluated using an Annexin V‐FITC and propidium iodide (PI) double‐staining assay (Annexin V‐FITC Apoptosis Detection Kit, Sigma‒Aldrich, Cat#APOAF). A549 cells were cultured and subjected to baseline control conditions (CM, *n* = 3) or serum deprivation (DMEM, *n* = 3), with a treated group serving as a positive control (cisplatin treatment, *n* = 1). Following treatment, cells were harvested, washed with cold PBS, and stained according to the manufacturer's instructions. Cells were acquired using flow cytometer (BD FACS Melody BD Biosciences), and data were analyzed using FlowJo software (Treestar Inc.) to distinguish viable cells (Annexin V^−^/PI^−^, Q4), early apoptotic cells (Annexin V^+^/PI^−^, Q3), late apoptotic cells (Annexin V^+^/PI^+^, Q2), and necrotic or advanced damaged cells (Annexin V^−^/PI^+^, Q1). Quantitative results are presented as mean ± standard deviation (SD).

### 5.4 LDH cytotoxicity assay

Cellular damage and cytotoxicity were evaluated by measuring LDH release into the culture supernatant using the LDH Cytotoxicity Assay Kit (Cat#MAK529) according to the manufacturer's protocol. Following the treatment of A549 cells under control conditions, serum deprivation (DMEM), or positive control treatment (cisplatin) (*n* = 3), cell‐free supernatants were collected and incubated with the assay reaction mixture. Absorbance was measured using a microplate reader (Multiskan SkyHigh, Thermo Fisher Scientific Inc.). Data are presented as mean ± SD.

### 5.5 qPCR‐based cfDNA quantification and fragment integrity analysis

cfDNA levels were measured using qPCR on a QIAquant 96 5plex (Qiagen, Cat#9003010) with absolute quantification derived from standard curve‐based estimation. cfDNA concentrations were normalized to the volume of input conditioned media and reported as ng/mL.

Quantification was performed using assays targeting human LINE‐1 (hLINE‐1) repetitive elements, which provide high sensitivity due to their genomic abundance.^49^ A short 82‐bp amplicon (forward 5′‐TCACTCAAAGCCGCTCAACTAC‐3′; reverse 5′‐TCTGCCTTCATTTCGTTATGTACC‐3′) was used for cfDNA measurement. Fragment integrity was evaluated using a 224‐bp amplicon (forward 5′‐TCTGCCTTCATTTCGTTATGTACC‐3′; reverse 5′‐TCAGCACCACACCACACCTATTC‐3′) in conditioned media samples, following previously established approaches for cfDNA fragmentation analysis.^50^ The fragment integrity was defined as the ratio of long to short amplicon abundance, providing a quantitative estimate of cfDNA fragmentation status.

qPCR reactions were carried out using Kapa SYBR Green Master Mix (Kapa, Cat#KK4601) following the manufacturer's recommended protocol.

### 5.5 Capillary electrophoresis analysis of cfDNA fragment size

cfDNA fragment size distribution was assessed by capillary electrophoresis using the Agilent 4200 TapeStation system (Agilent Technologies) with High Sensitivity D1000 ScreenTape and reagents (Agilent Technologies, Cat#5067‐5584), following the manufacturer's instructions. Samples were analyzed using Agilent TapeStation software.

### 5.6 Nanopore library preparation, sequencing and pre‐processing

The complete cfDNA recovered from each extraction was used for nanopore library preparation using the Oxford Nanopore Technologies ligation sequencing kit (ONT, Cat# SQK‐NBD114‐24) following the manufacturer's protocol with modifications to accommodate the low‐input cfDNA and improve retention of short fragments. The incubation time during end preparation/repair was increased to 30 min. During bead‐based clean‐up, a bead volume equivalent to three times the sample volume was used to enhance recovery of small cfDNA fragments. No PCR amplification was performed. Nanopore libraries were prepared separately for each independent cfDNA extraction, multiplexed, and sequenced on the MinION platform using R10.4 flow cells (ONT, Cat# FLO‐MIN114).

Sequencing was performed using MinKNOW (v25.09.16, Oxford Nanopore Technologies). Base calling was carried out in real time using Dorado (v7.11.2) with the super‐accuracy model (dna_r10.4.1_e8.2_400 bps_sup@v5.0.0). Multiplexed reads were demultiplexed assuming dual‐end barcoding (SQK‐NBD114‐24, –barcode‐both‐ends), and adapter and barcode sequences were trimmed before alignment step (–trim all). Reads were aligned to the human reference genome (GRCh38/hg38) using minimap2 (v2.28 (r1209)) integrated within Dorado. Alignment files were processed using samtools (v1.19.2), retaining primary mapped alignments, excluding unmapped, secondary and supplementary alignments. Base‐frequency matrices generated across the terminal 10‐bp regions of both read ends identified a pronounced technical bias at the terminal 3′ position, with adenine accounting for approximately 95% of bases across samples. This pattern was consistent with the enzymatic dA‐tailing step used during library preparation and was therefore excluded from downstream motif analysis. Reads were discarded if they exhibited significant soft‐clipping (>10% of total read length) or possessed terminal mismatches, insertions or end‐clips exceeding 1 bp (allowing a single clipped base to preserve valid reads carrying the expected 1‐bp dA‐tailing extension without losing genuine cfDNA fragments). For downstream motif extraction, 3′ sequence windows on forward reads were shifted inward by one base to skip the artificial 3′‐dA‐tailing site. Similarly, 5′ extractions on reverse reads were started at the second base to account for the tolerated single base end clip, ensuring that only genuine genomic terminal sequences were profiled. All downstream analyses were performed using all reads that passed the predefined quality control (QC) and alignment filters. To assess the robustness of the results to differences in sequencing depth, a sensitivity analysis was performed by randomly down‐sampling the QC‐passed reads from each replicate to a uniform depth matching the lowest sample (9710 reads), followed by repetition of the complete analysis workflow.

### 5.7 Fragment length analysis

Fragment lengths were derived from each mapped read using samtools and custom AWK scripts. Read length counts were normalized to per‐sample probability densities, and individual density profiles were consolidated. Length distributions were visualized as density profiles using Matplotlib (v3.10.0) and Seaborn (v0.13.2), with consistent axis scaling and annotation of nucleosomal fragment sizes.

### 5.8 Fragment‐end motif analysis

Strand‐aware 5′ end motifs (2‐mers and 4‐mers) were extracted and pooled from both ends of each fragment (End 1 and End 2) using custom AWK and Python scripts. For forward reads (SAM FLAG bit 16 = 0), End 1 motifs were extracted directly from the sequence start, and End 2 motifs were obtained by taking the reverse complement of the 3′ sequence tail.

Background genomic frequencies were calculated from the hg38 reference genome. Motif enrichment was computed as the ratio of observed to expected frequencies and expressed as log_2_‐transformed values.

### 5.9 Illumina sequencing and data analysis

To evaluate the technical robustness of cfDNA 4‐mer end‐motif profiles and eliminate potential platform‐specific biases, single replicates of each cell line (A549, H460 and WI38) were subjected to independent Illumina paired‐end sequencing. Paired‐end sequencing reads were aligned to the hg38 human reference genome using BWA‐MEM (v0.7.17). Following alignment, BAM files underwent, strand‐aware processing pipeline where properly paired primary alignments were extracted using samtools (‐f 2 ‐F 2304) to exclude unmapped mates, secondary alignments and supplementary reads. Reads with excessive soft‐clipping (>10%) or terminal artefacts were filtered out using custom awk scripts. Strand‐aware extraction of the first four terminal nucleotides was then performed, incorporating reverse‐complementation where appropriate to generate accurate 4‐mer frequency matrices.

### 5.10 Dimer‐level motif and motif ratio index

To reduce dimensionality, 4‐mer motifs were collapsed into 2‐mer representations based on the first two nucleotides, generating frequency profiles for all 16 possible dimers.

A exploratory motif ratio index was defined to summarize dimer‐level motif patterns:

$$\begin{array}{ccc} & & \mathrm{Motif}\;\mathrm{ratio}\;\mathrm{index}=\\ & & \frac{\mathrm{AT}+\mathrm{CT}+\mathrm{GT}+\mathrm{CG}+\mathrm{AG}}{\mathrm{CC}+\mathrm{GC}+\mathrm{GA}+\mathrm{GG}+\mathrm{AC}+\mathrm{TA}+\mathrm{TT}+\mathrm{TG}+\mathrm{TC}} \end{array}$$

### 5.11 Processing of publicly available clinical cfDNA datasets

Publicly available Oxford nanopore sequencing data of plasma cfDNA from seven healthy individuals and six lung cancer patients were obtained from the NCBI SRA (BioProject PRJNA768481), originally generated in a previously published study.^46^ Aligned BAM files were retrieved from the European Nucleotide Archive, and 5′ end motifs were extracted and quantified using the same procedure applied to the cell‐line data.

Publicly available plasma cfDNA sequencing data from patients (*n* = 50) with NSCLC were obtained from the SRA (BioProject: PRJNA642002). The dataset included samples harboring activating EGFR exon 19 deletions and exon 21 L858R mutations; corresponding SRA accession IDs are provided in Table S3.

FASTQ files were processed using fastp (v0.23.4) for adapter trimming and quality filtering, followed by alignment to the hg38 reference genome using BWA‐MEM (v0.7.17). Only properly mapped reads were retained for downstream analysis. Duplicate reads were removed using Picard (v3.0). Fragment‐end motif extraction and enrichment analyses were performed as described above. Deduplicated BAM files were used for mutation detection, and somatic variants were identified using GATK Mutect2 (v.4.6.2.0) in tumour‐only mode.

### 5.12 Estimation of ctDNA concentration

ctDNA concentration was estimated using VAF values corresponding to EGFR exon 19 deletions and exon 21 L858R mutations. Total cfDNA concentration was obtained from the SRA metadata for each sample. ctDNA abundance was approximated as:

ctDNA=VAF×totalcfDNAconcentration

### 5.13 LASSO regression analysis

The relative frequencies of all 16 possible 5′ dinucleotides were used as predictors of log_10_‐transformed ctDNA concentration. Features were standardized and analyzed using L1‐regularized linear regression (LASSO), with the regularization parameter selected by cross‐validation. Model performance was assessed using fivefold cross‐validation, with out‐of‐fold predictions generated for each sample. Pearson and Spearman correlations were calculated between observed and predicted ctDNA concentrations.

### 5.14 Statistical analysis

All analyses and data visualization were conducted in Python (v3.10) using pandas (v2.2.2), NumPy (v2.0.2), SciPy (v1.16.3), Matplotlib (v3.10.0), Seaborn (v0.13.2) and scikit‐learn (v1.6.1). Differences in central tendency (median fragment length) between extracellular compartments were assessed using a two‐sided Mann–Whitney *U*‐test. Similarity of motif enrichment profiles across samples and experimental conditions was assessed using Pearson and Spearman correlations, as appropriate, and distributional differences were quantified using JSD. Linear regression was used to evaluate concordance between conditions, with deviation of the slope from unity tested using a two‐sided *t*‐test. Differences in motif frequencies between groups were assessed using Mann–Whitney *U*‐tests or two‐sample Student's *t*‐tests, as appropriate, with multiple testing correction performed using the Benjamini–Hochberg method (FDR < .05). Cross‐platform concordance of 4‐mer motif profiles was assessed using Spearman rank correlation and Euclidean distance. Associations between the motif ratio and estimated ctDNA abundance were evaluated using Spearman and Pearson correlations.

### 5.15 Computational environment

All data processing and analysis were performed using Bash‐based pipelines in a Linux environment and Python‐based (v3.12.13) workflows in Google Colab. Custom scripts were developed for data processing, and all steps were applied uniformly across samples to ensure reproducibility.

## AUTHOR CONTRIBUTIONS

*Conception, design, study and development of methodology*: Dhruv Das, A.V.S.S. Narayana Rao and Rajani Kant Chittela. *Acquisition of data*: Dhruv Das, Prachi Gupta and Rahul Tambade. *Analysis and interpretation of data*: Dhruv Das, A.V.S.S. Narayana Rao and Rajani Kant Chittela. *Writing the manuscript*: Dhruv Das and Rajani Kant Chittela. *Review and edit of the manuscript*: P.A. Hassan, A.V.S.S. Narayana Rao and Rajani Kant Chittela. *Supervision*: A.V.S.S. Narayana Rao and Rajani Kant Chittela.

## CONFLICT OF INTEREST STATEMENT

The authors declare they have no conflicts of interest.

## ETHICS STATEMENT

Not applicable.

## Supporting information



Supporting information

## Data Availability

All healthy and patient sequencing data analyzed in this study are publicly available from the Sequence Read Archive (BioProject: PRJNA642002, BioProject PRJNA768481), with accession numbers provided in Table S3. Raw sequencing data from cell‐line experiments, along with processed data and derived matrices, are available from the corresponding author upon request.
