# Supplementary material for: Fragment‐end sequence architecture of cell‐free DNA and its association with tumour DNA in non‐small cell lung cancer
Source: Clin Transl Med. 2026 Nov 14;16(11):e70901. doi: 10.1002/ctm2.70901 (PMC13770214; doi:10.1002/ctm2.70901)
Supplement: Supplementary file 1 [file CTM2-16-e70901-s001.docx]

**Supplementary Figure S1.** **Characterization of extracellular cfDNA integrity and fragment sequence profiles.**

(A) Fragment integrity of cfDNA recovered from the conditioned media of A549, H460, and WI38 cells, measured using qPCR (mean ± SD, n = 3).

(B) Capillary electrophoresis profiles showing fragment size distributions of cfDNA. The profiles are displayed using dual y-axes (primary axis: A549; secondary axis: H460, WI38, and Ladder) to normalize for signal intensity differences while keeping the fragment size (x-axis) scale comparable across all cell types.

(C) Comparison of 4-mer end-motif enrichment scores (Full Depth). Grouped bar plots depict the relative enrichment (log₂ ratio of observed vs. genomic background frequency) for the conserved consensus 4-mer motifs across A549, H460, and WI38 samples. Motifs are ordered by enrichment level, with positive values indicating over-representation at fragment ends and negative values indicating depletion.

(D) Fragment length density distribution with standard deviation bands across down-sampled cell line replicates. Shaded lines illustrate mean fragment length density (± standard deviation) for A549, H460, and WI38 down-sampled samples across the 0–1000 bp range, with vertical dashed lines highlighting major nucleosomal landmarks (167 bp, 334 bp, and 501 bp).

(E) Correlation matrix comparing full-depth versus down-sampled cfDNA replicates across all 256 motifs, with corresponding Spearman correlation coefficients (ρ).

(F) Log₂ enrichment of shared enriched and depleted 4-mer end-motifs across down-sampled cell line replicates. Bar plots illustrate the mean log₂ motif enrichment (observed versus genomic background) for selected shared 4-mer motifs across A549, H460, and WI38 down-sampled datasets.

(G) Scatter plot showing 4-mer end-motif frequencies in WI38 cfDNA versus gDNA processed via identical sequencing pipelines, with corresponding Spearman correlation coefficients (ρ).

(H) Scatter plots comparing 4-mer end-motif enrichment ratios between the primary nanopore sequencing dataset (“_N”) and the Illumina cross-validation dataset (“_I”) for A549, H460, and WI38 cell lines. The dashed grey line indicates the identity line (y = x), the dark red line represents the linear trend, and text annotations display the Spearman rank correlation coefficient (ρ) and corresponding p-value for each comparison.

**Supplementary Figure S2. Characterization of extracellular fractions and cfDNA abundance, integrity and fragment sequence profiles.**

(A) Transmission electron microscopy images showing round to oval vesicles with diameters of approximately 100 nm. Scale bar represents 500 nm.

(B) Nanoparticle tracking analysis (NTA) showing the particle size distribution of isolated extracellular vesicles, with a predominant population centered around ~150 nm.

(C) Size distribution of isolated vesicles measured by DLS at 25 °C, with samples diluted in PBS. The plot shows the intensity-weighted hydrodynamic diameter distribution.

(D) Immunoblot analysis demonstrating enrichment of canonical exosomal markers CD63 and TSG101 in the exosome fraction.

(E) Immunoblot analysis demonstrating absence of the negative marker calnexin in the exosome fraction, with A549 cell lysate included as a positive control.

(F) Quantification of cfDNA abundance in extracellular fractions measured by qPCR. Statistical comparisons between compartments were performed using a paired t-test (**P < 0.01; *P < 0.05, ns: not significant).

(G) qPCR-based fragment size assay to estimate relative cfDNA integrity between compartments. Statistical comparisons were performed using a paired t-test (**P < 0.01).

(H) Fragment length distributions of cfDNA isolated from exosome-enriched (EXO) and exosome-depleted supernatant (SUP) fractions of H460 and WI38

(I-J) Scatter plots comparing motif enrichment ratios (relative to chromosomal background) between exosome-associated and supernatant cfDNA for H460 (I) and WI38 (J). Each point represents an individual 4-mer motif. Strong concordance of motif enrichment patterns is observed across compartments (H460, ρ = 0.83; WI38, ρ = 0.92).

(K) Volcano plot displaying differential motif enrichment between exosome-associated and supernatant cfDNA fractions, with the x-axis representing log_2_ fold change (exosome/supernatant) and the y-axis representing statistical significance (log_10_ adjusted p-value).

**Supplementary Figure S3.** (A) Annexin V-FITC/PI flow cytometry analysis of A549 cell viability under baseline, serum-deprived, and stress conditions. Representative scatter plots show cell populations divided into quadrants for viable cells (Q4, Annexin V⁻/PI⁻), early apoptosis (Q3, Annexin V⁺/PI⁻), late apoptosis (Q2, Annexin V⁺/PI⁺), and secondary necrosis/damaged cells (Q1, Annexin V⁻/PI⁺) under Control (CM, n = 3), serum-deprived (DMEM, n = 3), and Positive Control (cells treated with cisplatin, n = 1) conditions. Values represent mean ± SD.

(B) Quantification of relative LDH release following serum deprivation (DMEM) and positive control treatment (Cisplatin) compared to baseline controls in A549 cells (n = 3). Data represent mean ± SD

(C) Quantification of extracellular cfDNA released from A549, H460, and WI38 cells cultured under serum-containing (CM) or serum-deprived (DMEM) conditions. Serum deprivation significantly increased cfDNA abundance across all cell lines (all p < 0.005).

(D) Fragment length distributions of cfDNA isolated from complete medium (CM) and serum-deprived (DMEM) supernatant fractions of H460 and WI38 cells.

(E-F) Pairwise comparisons of normalized 4-mer fragment-end motif frequencies between serum-containing (CM) and serum-deprived (DMEM) conditions for H460 (E), and WI38 (F) cells. Each point represents an individual 4-mer motif.

**Supplementary Table S1.** Enrichment of 4-mer terminal motifs in extracellular cfDNA.

Enrichment values of 4-mer motifs at cfDNA fragment ends relative to genomic background frequencies. Values were calculated as log₂ (observed motif frequency ÷ genomic background frequency).

**Supplementary Table S2.** Differential abundance of 4-mer end-motifs between exosome-associated and supernatant fractions in A549 cells

**Supplementary Table S3**. Estimated circulating tumor DNA (ctDNA) abundance in NSCLC patient plasma samples.
